# Into the Unknown: The Shift in Key Service Performance Indicators after a Clinical Hospital Department Incorporates Virtual Service Delivery Options

**DOI:** 10.1055/s-0044-1781462

**Published:** 2024-03-04

**Authors:** Angela Vivanti, Eryn Murray, Ra'eesa Doola, Jan Hill, Clair Sullivan

**Affiliations:** 1 Department of Nutrition and Dietetics, Princess Alexandra Hospital, Brisbane, Queensland, Australia; 2 School of Human Movement and Nutrition Studies, The University of Queensland, Saint Lucia Campus, Saint Lucia, Queensland, Australia; 3 PA-Southside Clinical Unit, Faculty of Medicine, The University of Queensland, Brisbane, Queensland, Australia; 4 Queensland Digital Health, Centre for Health Services Research, The University of Queensland—Saint Lucia Campus, Brisbane, Queensland, Australia

**Keywords:** electronic health records, COVID-19, transmission risks, face-to-face and virtual service delivery

## Abstract

**Background** Coronavirus disease 2019 (COVID-19) forced health care services to introduce virtual service delivery. Little is known about the impact on health care service delivery.

**Objectives** This case study reports the impact of introducing remote access facilitating virtual service delivery.

**Methods** Key performance indicators of health care service delivery (Nutrition and Dietetic Department, 26.9 full-time equivalents) were monitored over three 6-month periods. These periods were Phase 1 (pre-COVID restrictions), Phase 2 (zero tolerance to COVID), and Phase 3 (living with COVID). Virtual service delivery was initiated between Phases 1 and 2. Virtual service delivery days were defined as days worked virtually in lieu of leave.

**Results** During Phase 2 and Phase 3, there were 87 and 188 extra days of virtual service delivery achieved and an opportunity cost saving of $26,000 USD and $56,000 USD, respectively. Leave hours reduced between Phases 1 and 2 ( *p* < 0.006; mean ± standard deviation [SD] 591 ± 213 and 222 ± 91) and maintained between Phases 1 and 3 ( *p* < 0.342; mean ± SD 494 ± 98) despite the pandemic. No adverse clinical events were reported. Professional quality of life scores were maintained.

**Conclusion** Virtual service delivery through remote access provided many days of otherwise potentially lost productivity, maintained patient care with no adverse events, and sustained Professional Quality of Life despite pandemic challenges. Operationally, lessons learnt included the importance of positive team culture to working effectively, keeping teams connected and adapting different solutions to meet teams' requirements. Incorporating virtual service delivery options into a hospital clinical department showed performance stability across key service performance indicators during the COVID-19 pandemic.

## Introduction

Coronavirus disease 2019 (COVID-19) has acted as a disruptor to conventional health care service provision. To maintain the safety of patients and health care workers, many health care providers introduced virtual models of clinical care in response to the pandemic.

Many hospitals adopted telehealth, minimizing nonessential attendance at the hospital during the initial peak of the COVID-19 pandemic, 1 2 with the addition of flexible work arrangements to minimize transmission risks. 3 4 A systematic review regarding health conditions and telehealth models demonstrated a slight reduction in hospitalizations, but no data have been reported on the impact of remote access upon staff leave and productivity. 5

Our hypothesis was that a shift to a hybrid model incorporating face-to-face and virtual service delivery within our department would not result in negative impacts on service delivery. Our aim was to investigate the impact of introducing a hybrid model on key performance indicators (KPIs) of health care service delivery pre- and post-implementation.

## Setting

This case study occurred in the Department of Nutrition and Dietetics (39 staff; 35/39, 90% female; 26.9 full-time equivalents) at a large academic hospital that has been awarded Electronic Medical Record Adoption Model Stage 6 recognition by the international Healthcare Information and Management Systems Society. As a major tertiary facility, staff provide clinical care to both acute and chronic inpatients and outpatients. Other activities delivered by staff include foodservice activities, research, teaching, and administration. Service delivery primarily occurs face to face; however, remote access to facilitate virtual service delivery was available for all staff unable to be on site but able to work including if they confirmed COVID-19, showed the presence of COVID-19 symptoms, waited for test results, or were in close contact. A detailed breakdown of all the applications used to facilitate virtual service delivery is outlined in Table 2 .

## Study Design

This is an observational study of impacts of a change to a hybrid model including virtual service delivery upon clinical health care service KPIs. Data were collated from parameters routinely captured within the department or organization over three different phases during the pandemic, in a duration of 6 months. These phases are as follows: (1) pre-COVID restrictions; (2) zero tolerance of COVID; and (3) living with COVID phase (detailed in Table 1 ). 6 This final phase was reached once 90% of the population was vaccinated. This timeline is represented below in Table 1 .

**Table 1:** Timeline showing study periods and dates and key COVID pandemic events

Phase 1	Pre-COVID-19 pandemic (face-to-face service delivery) June to November 2019
	WHO COVID-19 pandemic announcement 7 Australian national border closures with travel restrictions 8 Virtual service delivery initiated by department
Phase 2	Zero tolerance (virtual service delivery) June to November 2020
	State and national border closures with travel restrictions to Australia, 8 strict mask legislations and check-in applications
	State borders reopen to the remainder of the country
Phase 3	Living with COVID (virtual service delivery) January to June 2022
	Following state borders reopening to the remainder of the country. Continuing full requirement for masks and check-in applications until March.
	Ceasing requirement to check-in, wear masks (except with vulnerable populations, travelling on flights, shared or public transport) and be fully vaccinated in a range of venues and events 9
	National borders reopened once 90% population vaccinated. Requirements for masks and check-in applications ceased.

Abbreviations: COVID-19, coronavirus disease 2019; WHO, World Health Organization.

**Table 2:** Applications made available for virtual service delivery through remote access

Virtual access to applications independent of on-site computers		Accessing on-site computers remotely
Clinical applications	Administrative and contemporary applications	Direct remote desktop connection providing additional access to
Auslab (pathology application) ieMR (Cerner application) b Metaviasionintensive care unit c Viewer health provider portal	Adobe Acrobat Reader d Excel a MyHR (human resource platform) Notebook OneDrive a OneNote a Outlook a Powerpoint a Riskman (incident reporting system) e S4HANA (business management software) f SharePoint a Teams a Web browser (Microsoft Edge a , Internet explorer a ) Word a	Cbord (food service software) HBCIS-Queensland (hospital-based corporate information system for patient tracking) iLearn@QHealth (learning management system) LEAPOnline (online learning platform -replaced) MSHLearn (online learning platform - replacement) Patient Flow Manager (patient tracking system) Spok Messenger (paging service)

a Microsoft Corporation, Washington, United States.

b Cerner Missouri, United States.

c iMDsoft Isreal.

d Adobe Inc., California, United States.

e RLDatix London, United Kingdom.

f SAP Business Suite, Waldorf Germany.

### Intervention

Virtual service delivery was enabled by remote access to electronic health records, health department applications, and cloud-based team collaboration software ( Table 2 ). All staff within the department were provided with approvals and then assisted with the set-up of their remote access to workplace clinical, administrative, and contemporary applications between April and May 2020.

### Key Performance Indicators

The KPIs measured pre- and post-virtual service delivery are outlined in Table 3 .

**Table 3:** Key performance indicators investigated during clinical service provision using virtual service delivery

Key performance indicator (measure)	Definition
Leave required (hours) a	Sick leave or carer's leave (hours) when staff member unable to work due to illness or caring for immediate family or household members who are unwell.
Extra days work enabled by remote access (hours) a	Extra days of work delivered via virtual service delivery where previously leave would have been taken
Professional quality of life Scale 10 (raw scores)	Validated tool completed periodically measuring compassion fatigue, work satisfaction, and burnout in helping professionals
Riskman incident reporting (number)	Number of adverse incidents reported and investigated
Conservative estimated financial productivity gained	Top wage rate of entry level position 2021 $39.79 USD hourly 11 without addition of any oncosts 12 multiplied by hours not needing to be taken as leave

a Internal departmental attendance documentation.

### Data Collection

Sick and carer's leave amounts were sourced from the human resource leave system and reported as total leave. Days worked remotely, in lieu of accessing sick or carer's leave, were coded and recorded prospectively in the departmental attendance documentation. Hours were divided by 7.6 so equivalent to 1 working day. All adverse clinical events recorded by the organization were reviewed from organizational reports. Professional quality of life survey data were collated as part of usual departmental practice. Perspectives from the authors, who form part of the leadership group (A.V., E.M., R.D.), were collated on the “success factors” required to implement virtual service delivery.

### Statistical Analysis

SPSS for Windows, release 23.0 (Statistical Package for the Social Sciences; SPSS Inc., Chicago, IL, United States) was utilized. Descriptive data were presented as counts and percentages, while normally distributed continuous data were presented as mean and standard deviations. Continuous parametric variables were compared between groups using independent samples *t* -tests with statistical significance considered at *p* < 0.05.

## Results

**Fig. 1:**
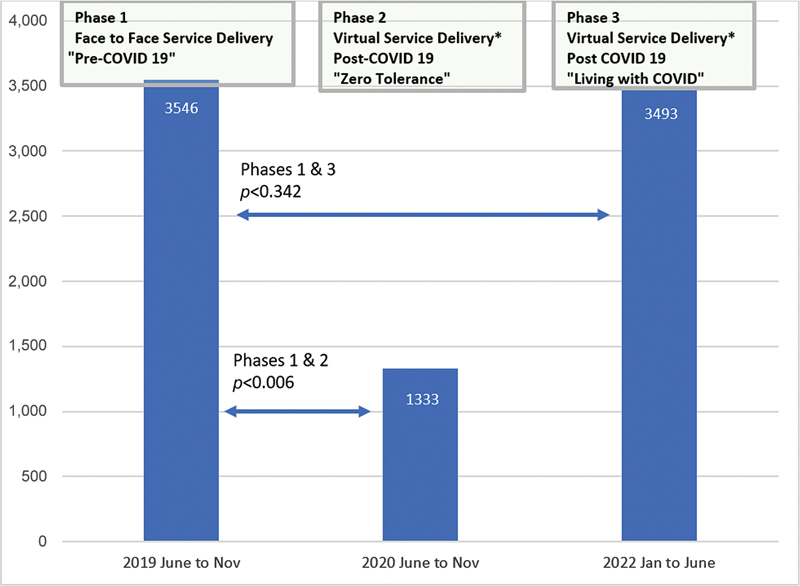
Hours of leave per phase pre- and post-virtual service delivery implemented due to the COVID-19 pandemic. *Virtual Service Delivery initiated April 2020.

Leave hours after introducing remote access enabling virtual service delivery are presented in Fig. 1 .

Following the introduction of virtual service delivery prior to phases 2 and 3, a statistically significant decrease in hours of leave monthly was evident between phases 1 and 2 ( *p* < 0.006; mean ± SD 591 ± 213 and 222 ± 91) and remained similar between phases 1 and 3 ( *p* < 0.342; mean ± SD 494 ± 98; Fig. 1 ) despite the worst pandemic in a century.

Over the 6-month periods, 87 and 188 days of virtual service delivery were provided in place of leave during phase 2 “zero tolerance” and phase 3 “living with COVID,” respectively ( Table 4 ). No adverse events related to the practice change were reported during this period.

**Table 4:** Productivity and virtual care

	Phase 1 face-to-face service delivery pre-COVID-19 2019	Phase 2 virtual service delivery b post-COVID-19 2020 “zero tolerance”	Phase 3 virtual service delivery b progression to “living with COVID” 2022
Days of leave taken a	455	171	447
Days worked providing a service through virtual delivery instead of requiring leave:	Not applicable	87	188
Proportion of days providing a service though virtual delivery instead of requiring leave	–	34% c	30% c
Conservative estimated financial productivity gain in 6 months	–	$26,309 USD	$56,561 USD

Abbreviation: COVID, coronavirus disease.

a Total hours were divided by 7.6 hours to represent work days within a 38-hour work week.

b Virtual service delivery initiated April 2020.

c Proportion calculated from the “days worked providing a service through virtual delivery” divided by the total of “days of leave taken” plus “days worked providing a service through virtual delivery instead of requiring leave.”

Virtual service delivery presented a productivity opportunity cost saving of over $56,000 USD during 6 months while progressing to “living with COVID” as demonstrated by the days of virtual service delivery instead of requiring leave.

High professional quality of life mean scores were maintained. Scores remained stable with no statistically significantly differences were evident in 2019 ( *n* = 24) prior to COVID-19 or in 2020 ( *n* = 11) during COVID-19 for compassion, burnout, secondary trauma, or total score (mean ± SD 2019 vs. 2020: 39.1 ± 5.8 vs. 40.9 ± 4.9, *p* = 0.383; 22.6 ± 5.4 vs 22.4 ± 4.6, *p* = 0.908; 19.7 ± 3.7 vs. 17.8 ± 3.7, *p* = 0.176; 81.4 ± 7.4 vs. 81.1 ± 5.4, *p* = 0.897). Group means before and during COVID-19 were favorable.

The authors reported success factors for virtual service delivery to include a high degree of trust, ability to connect at any time by phone or Microsoft Teams, clear plans on how different aspects of workloads would be managed remotely, a system partnering staff members working remotely with an on-site staff member to action any necessary face-to-face encounters or physical requirements of the role.

## Discussion

Virtual service delivery using remote access, electronic health records, and telehealth enabled additional productive clinical health care service provision while maintaining patient safety with no adverse events.

Many clinical services were converted to telehealth when social distancing standards were implemented within finite office spaces and with staff requiring COVID-19 testing and isolation. Clinicians providing virtual service delivery completed outpatient clinic and medical record-based activities, while clinician colleagues onsite completed clinical tasks requiring physical presence. Flexibility, coordination, and cooperation were required.

Despite the worst pandemic in a century, the department maintained sick and carer's leave similar to pre-COVID levels. The department reported higher leave requirements during the “living with COVID” period compared with “zero tolerance” but the proportion of staff who provided virtual service delivery in place of accessing leave remained similar to the previous study period resulting in 30% less leave than was previously experienced prior to the introduction of virtual service delivery options. It has been proposed that access to remote work can help attract staff, lower attrition and retain experienced staff desiring flexibility during various life stages. 13 The additional flexibility of remote access enabling virtual service delivery is speculated to have assisted with maintaining a high departmental professional quality of life throughout COVID-19 despite challenging times requiring resilience and recognized as impacting mental health internationally. 14 15 16

Virtual service delivery was confirmed to be feasible for a tertiary teaching hospital department. The workforce impacts from COVID-19 enabled the implementation of flexible service provision through digital transformation by rethinking the blend of technology, people, and processes to maintain hospital service. Slow adoption of flexible work and service delivery in hospitals appears to represent an opportunity cost for the hospital sector. Other industries have speculated that not everyone desires to work remotely or is adequately disciplined to work in this fashion. 13

In the authors' experience, appropriate culture and management support were necessary for the successful implementation of virtual service delivery. Studies suggest more success with working from home arrangements where there is high trust and a lower power differential. 17 Work styles deemed most suitable for working from home included the extremes of work autonomy; from highly self-motivated and self-directed endeavors to the completion of tasks and activities which can be easily assessed and tracked. 13

The leadership group has reflected on the lessons learned and success factors and this is published elsewhere. 18 These included (1) the access available to digital health care through the electronic health record and the telehealth services; (2) the value of a preexisting positive and functional team culture; and (3) different teams (including acute, rehabilitation, and outpatient services) adapting different solutions to meet their teams' differing requirements. Strategies were implemented to ensure teams remained connected. Active regular communication within clinical teams may include virtually meeting at the start of day to organize team plans and revise patient allocations depending on individuals off-site, near lunch breaks to check progress and adjust plans as needed and then at the end of day to ensure all essential final tasks completed with whole team assisting. Teams completing greater outpatient services included virtual meal breaks for connectivity. Good professional quality of life was maintained during the extended period of the COVID-19 pandemic.

This opportunity highlighted avenues for increased flexibility in the way tertiary health care is delivered, supporting flexible working arrangements while ensuring service delivery. Certain aspects may be better suited to different specialties. We incorporated reallocation of tasks and a greater use of virtual meeting platforms both on and off sites, greater utilization of phone and telehealth consultation, and remote access to electronic health records. Components were utilized by teams as best suited their work requirements.

Virtual service delivery enabled through remote access considerably reduced leave requirements during a once in a century pandemic without adversely impacting patient outcomes or staff satisfaction. This sustainable service demonstrates tangible benefits and may assist in reducing burn out and maintain service delivery.
